# A pilot study of the moderating effect of gender on the physical activity and fatigue severity among recovered COVID-19 patients

**DOI:** 10.1371/journal.pone.0269954

**Published:** 2022-07-13

**Authors:** Monira I. Aldhahi, Mohammed M. Alshehri, Faleh Alqahtani, Abdulfattah Saeed Alqahtani

**Affiliations:** 1 Department of Rehabilitation Sciences, College of Health and Rehabilitation Sciences, Princess Nourah bint Abdulrahman University, Riyadh, Saudi Arabia; 2 Physical Therapy Department, Jazan University, Jazan, Saudi Arabia; 3 Medical Research Center, Jazan University, Jazan, Saudi Arabia; 4 Department of Pharmacology and Toxicology, College of Pharmacy, King Saud University, Riyadh, Saudi Arabia; 5 Department of Rehabilitation Sciences, College of Applied Medical Sciences, King Saud University, Riyadh, Saudi Arabia; Lluita contra la SIDA Foundation - Germans Trias i Pujol University Hospital, SPAIN

## Abstract

**Background:**

Clinical data point toward gender-based differences in COVID-19 severity. However, there is insufficient research examining whether gender predicts physical activity (PA) and fatigue severity in patients recovering from COVID-19. Therefore, this study aimed to characterize the PA and fatigue severity in a cohort of patients recovering from COVID-19 infection and measure the extent to which gender-based differences moderate the relationship of PA with fatigue.

**Method:**

A cross-sectional survey was conducted in Riyadh, Saudi Arabia. The sample comprised patients recovering from COVID-19 over at least 3 months. Recovered patients were stratified into two groups based on gender. The survey included items pertaining to sociodemographic, a fatigue severity scale and a self-reported international PA questionnaire.

**Results:**

Eighty-seven patients (44 women and 43 men) met the inclusion criteria. Compared with men, women reported sedentary behavior (70%) and high fatigue severity (64%). A significantly higher number of women had a low PA score compared with men (*p* = .002). The findings indicated that gender significantly moderates the effect of total PA in metabolic equivalents (METs; min/wk) on fatigue severity [*F* = 4.8, *p* = .03, ΔR^2^ = 0.24].

**Conclusions:**

The current study suggests that women might be at risk of higher fatigue severity, in addition to engaging less in PA. Physical activity may plays a significant role in modulate the fatigue severity. Consequently, interventions aimed at promoting physical activity in women stand high chances of addressing the disparity in the distribution of prevalence of fatigue between men and women.

## Introduction

The rapid and unprecedented outbreak of the novel coronavirus disease 2019 (COVID-19) has caused staggering global demands on all aspects of life around the world, including health and the economy. Accordingly, the intersectoral collaboration between health and non-health-related sectors has resulted in coordinated efforts to promote health and control the spread of the virus. Public health systems and healthcare providers have made concerted efforts to promote regular physical activity (PA) and improve health among those recovering from COVID-19 [1–4]. Engaging in PA after recovering from COVID-19 is essential for good health [3]. Over the last few decades, scientific studies have highlighted the plethora of health benefits of being physically active, ranging from cardiovascular to psychological wellbeing [5, 6]. These positive effects of PA are significant because they may help patients overcome cardiopulmonary sequelae and physical fatigue induced by COVID-19 [2]. It is recommended to start a phased approach to gradually resume PA at least seven days after contracting COVID-19 or once the patient is symptom free and risk stratified [7].

Patients recovering from COVID-19 have been known to suffer from physical fatigue, which can vary according to the severity of the prognosis. In light of new scientific evidence, it has been reported that COVID-19 affects patients’ physical wellbeing with symptoms that include increased fatigue, decreased fitness, and even psychological sequelae in some patients [8–10]. The fatigue concept introduced in this study is defined as “a subjectively unpleasant symptom that incorporates total body feelings ranging from tiredness to exhaustion, creating an unrelenting overall condition which interferes with individuals’ ability to function at normal capacity” [11]. This type of fatigue is a frequently debilitating symptom that strongly predicts a decline in PA and performance [12–14]. The fatigue–physical activity association is multifactorial, which can be explained by body composition, physical fitness, and comorbidity [15]. Thus, the vulnerability to fatigue from PA could be dependent on nutritional supplements with antioxidant properties, which may contribute to the severity of the fatigue [16, 17]. It is known that minerals and vitamins are essentials to avoiding fatigue and loss of energy [18]. Therefore, controlling for these factors in fatigue severity studies is required.

Although there is a body of knowledge on the association between fatigue and PA, the complex relationship between these factors and gender is limited. Moreover, although there is little known about the relationship between fatigue and PA levels in patients who have recovered from COVID-19, studies have revealed the nature of this relationship in several disease populations [19–21]. Clinical data regarding COVID-19 point towards gender-based differences in COVID-19 severity [22]. Furthermore, it has been shown that men experience a higher mortality and increased severity of COVID-19 than women [22–24]. Accordingly, the disparities experienced in recovering to a normal lifestyle post-COVID-19 infection require further exploration. Evidence of sex-based differences in immunological responses [22], gender-based differences in smoking behaviors [25], and PA [26] has been established. It is noteworthy that many factors can influence the PA and fatigue post-COVID-19 infection. One of these factors is gender, as it has been suggested that men may engage in higher PA levels [27]. These findings suggest the existence of gender-based differences in the PA patterns and fatigue severity among patients who have recovered from COVID-19. As returning to recommended PA after recovering from COVID-19 is essential. Hence, understanding gender sensitivities is a crucial component in creating effective regimen to improve PA behaviors.

Previous research has demonstrated a gender gap in physical activity and exercise motivation [28, 29], with women being less active and less inclined than men to engage in PA that can be done independently. However, few studies have examined whether this gender predicts PA and fatigue severity. Moreover, to the best of our knowledge, limited studies have examined the effects of gender interactions on the association between fatigue and PA. In light of this evidence, this study aims to examine the differences in PA pattern and self-reported fatigue severity in a cohort of patients who recovered from COVID-19 infection. Another aim of this study is to assess the moderating effect of gender on the association between fatigue and PA. We hypothesized that gender may moderates the relationship between the levels of PA and fatigue severity among patients who have recovered from COVID-19 infection.

## Materials and methods

### Study design and participants

This cross-sectional study was conducted in Riyadh, Saudi Arabia, between January and April 2021. The sample of this research study comprised 100 patients recovering from COVID-19. A list of the names of the recovered patients was obtained from the Ministry of Health in Riyadh via a non-probabilistic convenience sampling process. The study included patients who were confirmed to have COVID-19 in October 2020. The inclusion criteria were participants who were at least 18 years of age, had no history of cardiovascular disease associated with COVID-19, and had recovered from COVID-19 three months after the onset of infection. Recovered patients were stratified into two groups based on gender. Patients were excluded from the study if they had physical disabilities, a history of cardiovascular disease, or were admitted to the intensive care unit after contracting COVID-19. A total of 4 patients (three male and one female) were excluded from the analysis as they had severe comorbidities and were admitted in the ICU (> 5 days). In this research study, the patients were contacted through phone interviews; a total of 87 of the patients agreed to participate and completed the study. The total length of stay at the hospital among the participants range between 2–3 days.

### Ethics approval and consent to participate

The study was ethically approved by the Central Institutional Review Board (IRB) of the Ministry of Health (20-11E), Riyadh, Saudi Arabia. Prior to data collection, the study procedures and participants’ rights were explained, and informed consent was obtained. All respondents were asked to provide informed consent, which was obtained before they completed the survey. The information and responses were treated as confidential and anonymous.

### Instrument

The survey was generated using an online survey system. The first section consisted of questions pertaining to the participants’ sociodemographic, general health, and nutritional characteristics. Participants completed a questionnaire with queries related to the supplementary intake of vitamin B12, vitamin C, vitamin D, Omega−3 fatty acids, and Zinc (S1 Appendix). A pilot study using Cronbach’s alpha was conducted among a group of 30 participants to examine the reliability of the nutritional questions about supplementary intake; the results yielded good reliability (alpha (α) = 0.79). In addition, smoking status was obtained with a yes/no question. For anthropometric assessments, patients’ heights and weight were self-reported and recorded in centimeters, and body mass index (BMI) was calculated as weight divided by height squared (kg/m^2^).

In the second section, the fatigue severity scale (FSS) was used [30]. This is a self-reported questionnaire composed of a 9-item scale that rates the perceived severity of fatigue among adults in terms of how fatigue affects PA, activities of daily living, and motivation [31]. The scale was reported to have an acceptable internal consistency with a Cronbach’s alpha of 0.88. The Arabic version of the scale was used in this study, it was validated and found to have good internal consistency [31]. Psychometric properties of the Arabic version of the FSS scale showed acceptable test-retest reliability and internal consistency (intraclass correlation coefficient (ICC) = 0.80; Cronbach’s alpha = 0.84) [30]. Concurrent validity of the FSS was reported to have a good correlation with both the visual analog scale scores (*r* = -0.76) and vitality domain of Short-Form 36-item (*r* = -0.49) [32]. The responses used a 7-point Likert scale (“1 = strongly disagree” to “7 = strongly agree”). The FSS composite score was calculated by taking the average of the nine items. For healthy individuals, the mean FSS score was 2.3 and the standard deviation (SD) was 0.7. Previously established in the literature that a cutoff point of the FSS score greater than 4.05 indicated high fatigue severity [32].

The third section, PA was assessed using a short form of the self-reported international PA questionnaire (IPAQ-SF). The questionnaire was designed by Craig et al. [33] and comprises four generic items that reflect three types of intensity of PA (vigorous, moderate, and walking) and sitting time performed in the last 7 days. The Arabic version of the questionnaire was validated previously, and this version was used in the present study [34, 35]. The IPAQ-SF showed high stability and good internal consistency (Cronbach alpha = 0.75) [33].

The metabolic equivalents (i.e., METs/week) related to vigorous PA, moderate-intensity PA, and walking, and total time of PA performed were calculated using guidelines for data analysis [36]. PA was stratified into the following three levels: low, moderate, and vigorous. A high level of PA is defined as the individual’s ability to achieve PA of any combination of vigorous, moderate, or walking that equivalent to at least 3,000 METs per week. A moderate level of PA is defined as the individual’s ability to achieve PA equivalent to at least 600 METs per week. A low level of PA means that individuals are not meeting the criteria of vigorous or moderate levels of physical activity per week (PA is lower than 600 METs per week).

### Statistical analysis

The collected data were analyzed using Stata version 16 (StataCorp, College Station, Texas, USA). Data were examined to assess the normality assumption using the Shapiro–Wilk test. Descriptive statistics of frequencies and percentages are utilized to present demographic and categorical data. The median [interquartile range (IQR)] were reported for non-normally distributed variables. The analysis was conducted in two phases. The first phase was carried out to examine the gender-based differences in the main constructs of the study which are the fatigue severity and physical activity levels as dependent variables and gender as independent variable. Gender-based differences in PA and fatigue severity were examined using the Mann–Whitney U test since the assumption of normality was violated. The categorical variables of demographic characteristics were analyzed using Chi-square tests.

Forward stepwise linear regression was carried out, in which the total PA and fatigue severity were the dependent variables and gender as independent variable. In step 1, Model 1 was adjusted for age, smoking, marital status, and BMI; Model 2 was adjusted for Model 1 variables plus vitamin B12 supplement intake, vitamin C supplement intake, omega-3 supplement intake, and zinc intake. A simple moderator regression analysis, using the PROCESS macro of the Statistical Package for the Social Sciences, SPSS, version 3.5.3 developed by Hayaes [37], was done to assess the effect of gender as moderating variables on the relationship between fatigue severity and PA. Statistical significance was set at a *p*-value of less than .05.

## Results

### Demographic characteristics of the participants

Of the 100 patients diagnosed with COVID-19 in the indicated period, 87 patients met the inclusion criteria and consented to participate in the study. The demographic characteristics of the 87 participants who had recovered from COVID-19 are displayed in Table 1. The median ages of the participating men and women were 33 and 36 years, respectively. Most of the women (64%) and men (88%) were married, with no significant difference between the groups (*p* = .06). The proportion of smokers was significantly higher for men compared to women (67% vs. 0%, *p* < .001). Table 2 displays the descriptive statistics of dietary supplements that were taken during COVID-19 recovery.

**Table 1:** Demographic and general health characteristics of the participants.

Variables	Women n = 44	Men n = 43	*p*-value
**Age (years)**	33 (26–37)	36 (32–44)	.1
**Height (cm)**	159 (155–164)	170 (167–176)	< .001
**Weight (kg)**	67 (58–84)	79 (72–90)	.007
**BMI (kg/m** ^**2**^ **)**	27.5 (21–33)	26.9 (25–30)	.9
**Marital status, [n (%)]**			.06
Single	14 (32)	5 (12)	
Married	28 (64)	38 (88)	
Separated/divorced	1 (2)	0	
Widow	1 (2)	0	
**Educational status, [n (%)]**			.66
Bachelor’s degree	20 (45)	20 (47)	
Postgraduate degree	2 (5)	1 (2)	
Secondary school diploma	16 (36)	19 (44)	
Less than diploma	6 (14)	3 (7)	
**Employment status, [n (%)]**			< .001
Employed	24 (54)	30 (69)	
Unemployed	20 (45)	13 (30)	
**Smoking status, [n (%)]**			< .001
Smoker	0	29 (67)	
Non-smoker	44 (100)	14 (33)	
**Depression or anxiety, Yes [n (%)]**	0 (0%)	1 (2%)	.33
**Obesity, Yes [n (%)]**	2 (5%)	9 (21%)	.13
**Hypertensive, Yes [n (%)]**	3 (1%)	3 (7%)	.92
**Diabetic, Yes [n (%)]**	2 (5%)	6 (14%)	.16
**Older than 65 years, Yes [n (%)]**	0 (0%)	1 (2%)	.33
**Pulmonary disease Yes [n (%)]**	7 (16%)	3 (7%)	.14

Data presented as median [MED] (interquartile range [IQR]), Frequency [n] (percentage [%]); Mann–Whitney U test was used.

Abbreviation: Body Mass Index (BMI)

**Table 2:** Gender-based comparison of vitamin and dietary supplement.

Variables	Women n = 44	Men n = 43	*p*-value
**Omega-3 supplements,** Yes [n (%)]	2 (5%)	3 (7%)	0.7
**Vitamin C,** [n (%)]			.04
Don’t use	29 (66%)	16 (37%)	
Sometimes	9 (20%)	21 (49%)	
Always	6 (14%)	6 (14%)	
**Vitamin D,** [n (%)]			.2
Don’t use	30 (68%)	28 (65%)	
Sometimes	4(9%)	7 (16)	
Always	10 (23%)	8 (19)	
**Vitamin B12 supplements,** [n (%)]			
Don’t use	33 (86%)	29 (70%)	.05
Sometimes	0	6 (14%)	
Always	6 (14%)	7 (16%)	
**Zinc,** [n (%)]			
Don’t use	16 (36%)	10 (23%)	.5
Sometimes	22 (.50%)	29 (68%)	
Always	6(14%)	4 (9%)	

Data presented as Frequency [n] (percentage [%])

### Comparison of the PA pattern and FSS results

Table 3 shows the differences in PA patterns between men and women. The proportion of women reported sedentary behavior (70%) and high fatigue severity (64%) compared to the proportion of men who reported sedentary behavior (30%) and fatigue severity (28%). A significantly higher number of women had a low PA score on the IPAQ-SF compared with men (Pearson chi-squared (χ^2^) = 12.1; *p* = .002). Most of the men (79%) were classified as having moderate to vigorous levels of PA post-COVID recovery (Table 3). The changes in the proportion of PA level pre-COVID-19 and post-COVID-19 recovery were not statistically significant for either gender (women, *p* = .25; men, *p* = .36).

**Table 3:** Gender-based comparison of physical activity levels and fatigue severity.

Variables	Women n = 44	Men n = 43	*p*-value
**Vigorous PA (min/day)**	10 (0–30)	30 (0–65)	.006
**Moderate PA (min/day)**	15 (0–30)	40 (0–65)	.002
**Walking (min/day)**	20 (10–33)	30 (20–60)	.02
**Sitting (h)**	3 (2–5)	3 (1–4)	.6
**Total activity (min/wk)**	60 (20–90)	120 (50–190)	.001
**Total days of PA**	6 (3–7)	7 (4–7)	.001
**Vigorous PA METs (min/wk)**	0 (0–640)	960 (0–2160)	.003
**Moderate PA METs (min/wk)**	120 (0–480)	480 (0–1200)	.002
**Walking METs (min/wk)**	198 (56–536)	495 (297–990)	.008
**Total METs (min/wk)**	809 (149–1404)	1836 (990–4830)	.002
**PA categories before COVID-19,** [n (%)]			
Low	28 (64)	25 (58)	.05
Moderate	9 (20)	3 (7)	
Vigorous	7 (16)	15 (35)	
**PA categories after recovery from COVID-19,** [n (%)]			.002
Low	21 (48)	9 (21)	
Moderate	16 (36)	13 (30)	
Vigorous	7 (16)	21 (49)	
**Sedentary,** [n (%)]	7 (70)	3 (30)	0.1
**FSS scores**	4.1 (2.8–5.4)	2.4 (1.8–4)	.0002
**Fatigue categories,** [n (%)]			.001
Low	16 (36)	31 (72)	
High	28 (64)	12 (28)	

Data presented as median [MED] (interquartile range [IQR]), Frequency [n] (percentage [%]); Mann–Whitney U test was used. Abbreviation: Fatigue severity scale (FSS), Physical activity (PA), Metabolic equivalents (METs).

A total of 22 participants met the recommendation of combined moderate-to-vigorous PA each week (≥150 min/week), in accordance with the global recommendation of the World Health Organization [5]. Of these 22 participants, 16 were men (73%) and only 6 were women (27%). There was a significant difference between the proportions of men and women who met the recommendation (*p* = .01). The median time of vigorous intensity PA among men was triple that of women (30 vs. 10 min/day, *p* = .006). The median moderate intensity of PA time was reported to be significantly lower in women (*p* = .002).

The proportions of men and women above and below the cutoff point for fatigue severity are displayed in Table 3. Compared with men, there was a significant difference in the proportions of women who had greater fatigue (*p* = .001). While the female participants reported a high median FSS score of 4.1 (in a range of 2.8–5.4), the male participants reported a composite FSS score of 2.4 (in a range of 1.8–4) which was within the normal range of healthy individuals.

### Association of fatigue and PA severity with gender

The results of multiple linear regression analyses for the association between gender, fatigue severity, and total METs of PA are shown in Tables 4 and 5, respectively. Gender is considered a significant independent predictor of total PA and fatigue severity (*R*^2^ = .17, *p* = .003; *R*^2^ = .34, *p* = .006), respectively. This suggests that the total level of PA may be influenced by gender; female gender associated with fatigue severity. This could explain the 34% variance in the participants’ fatigue severity and the 17% variance in the participants’ total PA.

**Table 4:** Multiple linear regression analysis to assess the association of fatigue severity with gender as an independent variable.

Predictors	ß	95% CI		F	*R* ^2^	*p*-value
		LL	UL			
**Gender** ^**a**^	798.2	−3108.7	−498.6	10	.15	.002
**Gender** ^**b**^	2105.6	742.6	3468.5	9.5	.17	.003

note: reference group (male)

**a** Model 1 adjusting for (age, smoking, marital status and BMI)

**b** Model 2 adjusted for model 1 variables plus vitamin B12 supplements intake, vitamin C supplement intake, omega-3 supplement intake, and zinc intake).

Abbreviations: β = standardized beta; CI = confidence interval.

**Table 5:** Multiple linear regression analysis to assess the association of total pa with gender as an independent variable.

Predictors	ß	95% CI		F	*R* ^2^	*p*-value
		LL	UL			
**Gender** ^**a**^	−1.1	−1.8	−.3	4	.26	0.003
**Gender** ^**b**^	−1.2	−2.0	−.3	8	.34	.006

note: reference group (male)

**a** Model 1 adjusting for (age, smoking, marital status, and BMI).

**b** Model 2 adjusted for model 1 variables plus vitamin B12 supplement intake, vitamin C supplement intake, omega−3 supplements intake, and zinc intake).

Abbreviations: β = standardized beta; CI = confidence interval

**Fig 1:**
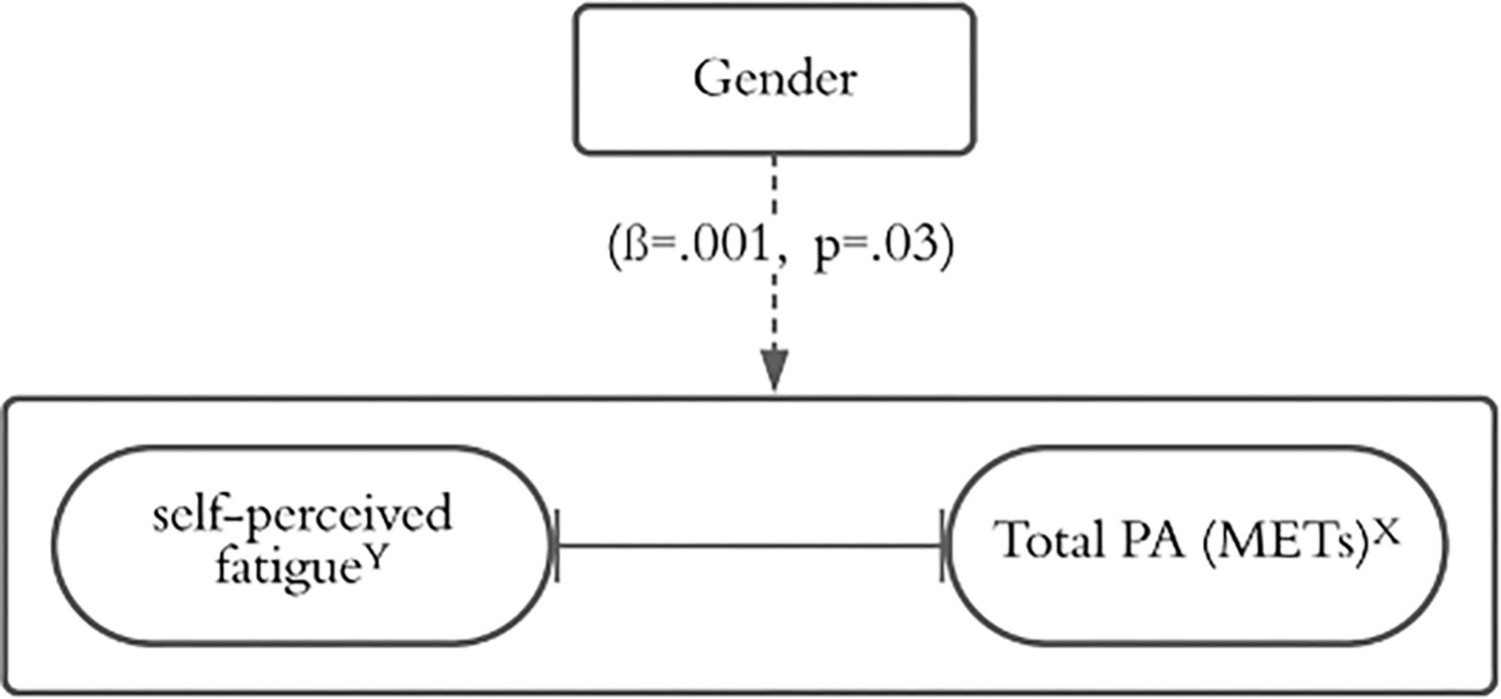
Moderating effect of gender on the relationship between self-perceived fatigue and moderate related PA (^Y^ denotes the dependent variables; ^x^ denotes the independent variables).

Further analysis was conducted to assess the moderating effect of gender on the relationship between PA and fatigue severity; a simple moderator regression analysis was done using PROCESS. The potential moderator for the analysis was gender, whereas fatigue was a dependent variable, and METs (min/week) of total PA represented the independent variable. Gender moderates the relationship between self-perceived fatigue and total PA (Fig 1). The intercept revealed a significant effect [*F* (1, 84) = 4.8, *p* = .03, ΔR^2^ = 0.24], suggesting a moderating effect of gender on the relationship. The interaction term was statistically significant (*β* = .001, *SE* = .006, *p* = .03), which suggests that gender is a moderator of the effect of total PA on fatigue severity. The effect of moderate PA on fatigue was negative and significant (*β* = -.002, *SE* = .001, *p* = .01), and the gender effect was negative and significant (*β* = -.1.4, *SE* = .39, *p* = .0005). These finding indicates that gender adds 24% extra variance in the association of PA with fatigue severity.

## Discussion

The findings of this study indicate that among patients who have recovered from COVID-19, women are more likely to report severe perceived fatigue compared to men. Furthermore, the findings of the current study revealed that gender moderates the relationship between self-perceived fatigue and total PA. Our findings are consistent with the results reported by Stavem et al., who found that 55% of female participants reported high fatigue (compared to 35% of male participants) at four months after the onset of COVID-19 infection [38]. Although the results are similar, Stavem et al. recruited a larger sample (N = 458) of older aged subjects (mean age = 49.6 years) and employed the Chalder fatigue scale [38]. They also found that female gender was associated with increased likelihood of fatigue after adjusting for age, sex, education, marital status, BMI, number of self-reported comorbidities, smoking status, history of depression, number of symptoms during acute COVID-19, dyspnea during COVID-19, and time since being diagnosed with COVID-19 diagnosis [38]. On the other hand, the finding of this study explores the moderating effect of gender on the relationship between physical activity and fatigue to support and expand on the findings of study by Stavem et al.

It should be noted that emerging review data suggest that the fatigue is considered a predominant symptom that persisted for months post COVID-19 infection. A systematic review of 18 perspective studies assessed the rate of fatigue associated with COVID-19 infection at various time windows, revealing that the fatigue rate ranged from 9–42% at 8–11 weeks and 32% at 28 weeks from the onset of symptoms [39]. In our sample, 45% had a high score for fatigue, which is consistent with the systematic review of the chronic phase [39]. Moreover, another study with a large sample size (2,113 participants, 85% women) reported fatigue as one of the most common symptoms presented three months after the onset of COVID-19, affecting 87% of the participants [40]. However, fatigue was self-reported with a yes/no question in this study (with no fatigue severity scale), and most of the participants were women. In addition, the researchers included all participants who were diagnosed with COVID-19, with the diagnosis being symptom-based [40]. In another study, Augustin et al. reported that female participants were associated with a higher risk of post-COVID syndrome, which consists of fatigue, anosmia, ageusia (a complete loss of taste function of the tongue), or shortness of breath. This was for people with mild symptoms four months after the onset of COVID-19 [41]. However, they did not utilize a validated method to diagnose fatigue. Instead, fatigue was determined only via a checklist of self-reported symptoms. The findings of the study emphasized the need to design individualized programs to overcome fatigue and its consequences among women. Thus, a prospective study that considers gender sensitivity and the severity of COVID-19 symptoms (using a biomarker of fatigue) is needed in the future to assess fatigue levels among COVID-19 survivors.

Based on emerging literature, there are some potential reasons may explain the prevalent of fatigued among women. First, women are at a higher risk of psychological disorders during the COVID-19 pandemic, which is a predictor of fatigue [42, 43]. In addition, COVID-19 can adversely affect skeletal muscles [44], which in healthy women have been found to be more prone to fatigue in healthy women to men [45]. Rudroff et al. [44] explained some potential contributing factors for this persistence fatigue, which include peripheral (e.g., changes in neurotransmitter levels and neurons inhibition) and psychological factors (e.g., stress, anxiety, and depression). Although the patients included in this study satisfied diagnostic criteria for post-infection fatigue syndrome (PIFS) [46], a careful clinical characterization of post-COVID19 fatigue is required to apply appropriate interventions. Further study is recommended to explore the mechanisms of fatigue and to classify the fatigue types among patients post- COVID-19 infection.

Our findings revealed differences in PA patterns between genders among people who have been infected with COVID-19. Women reported a high percentage of low levels of PA compared to males, who mainly reported a high percentage of vigorous levels of PA. The results of this study highlight the need to improve PA and fitness among women, because elevating cardiopulmonary fitness and levels of PA may help reduce the risk of various infections (including COVID-19) and improve immune biomarkers [47]. Some potential factors may contribute to these differences in PA, one of which is the inflammatory response in particular tumor necrosis factor alpha (TNF-α) is being reported to be significantly lower in women after severe COVID-19 infection than in men [48]. A systematic review indicated that genetic factors (single nucleotide polymorphisms), TNF-α, interleukin 1b, interleukin 4, and interleukin 6 are associated with all subgroups of fatigue [49]. These associations may play a role in low PA because they generate a sense of fatigue. The provided information about inflammatory pathway among women in this study was not investigated, and beyond the scope of the study. However, these factors may establish causation and explain the low level of physical activity among women compared with men who had recovered from COVID-19. Therefore, there is a need for more research efforts to address this topic.

The PA guidelines from the American College of Sport Medicine (ACSM) recommend that people exercise for at least 30 minutes at moderate intensity (or 20 minutes at vigorous intensity) for at least five days per week. Globally, it has been shown that women are less active (around 31.7% are inactive) than men (around 23.4% are inactive) [50]. Our data suggested that male COVID-19 survivors met the guidelines for performing moderate and vigorous activities, whereas female survivors were below the recommended cutoff scores. In line with our findings in Saudi Arabia, data from the General Authority for Statistics, in which 13 administrative regions and 26,000 respondents across Saudi Arabia were surveyed, indicated that the percentage of PA practice among women was lower (8.9%) than that of men (28.3%) [51]. Logically, the effects of the pandemic and the duration of lockdown in Saudi Arabia would influence both genders equally. However, several factors could explain the discrepancies in PA levels between genders. For example, women may be more anxious about participating in recreational activities due to fear of COVID-19 reinfection. A previous study showed more anxiety symptoms in women (25.1%) than in men (17.9%), and a higher prevalence of COVID-19 anxiety for women (24.6%) compared to men (17.7%) [52]. However, no anxiety was reported in this study, which excluded anxiety as an extrinsic factor. In addition, biases in the self-perception of the feminine or masculine traits of a given PA might have an effect on self-reporting, suggesting that further investigation and more objective measurements of PA are necessary [52].

The difficulties encountered when performing exercises in groups because of social distancing were highlighted by the ACSM as a primary concern related to meeting the PA guidelines during COVID-19 [53]. A systematic review study included 41 studies that aimed to identify the evidence related to PA during COVID-19 pandemic confirmed that social distancing measures were the main factors that caused low levels of PA [54], which could affect the participation and motivation levels of women engaging in activities. However, for the respondents in this study, the rate of regular PA engagement prior to COVID-19 infection was not significantly different from current PA engagement. Gender sensitivity in PA can be mediated by psychological and sociocultural factors. In a large database, it has been shown that social norms associated with parenthood and marital status were significantly influenced PA participation [55]. Despite several initiatives, self-perceptions of the feminine or masculine traits associated with a given activity and self-image may contribute to low levels of engagement in moderate-to-vigorous PA due to sociocultural norms in Saudi Arabia. Additionally, female role responsibilities in Western culture (such as taking care of the family and housework) could elicit challenges that control any decisions to exercise [26, 56]. However, the extent to which recovering from COVID-19 may result in changes to PA levels in women or men is still under investigation.

In the present study, a relationship was revealed between fatigue and PA, in which lower PA levels drive changes in the severity of fatigue. Moreover, gender appeared to moderate the relationship between PA and fatigue, which could be attributed to the different distribution of PA levels between men and women. Some studies have shown that more men engage in PA than women. For example, a study by Nienhuis and Lesser explored the impact of COVID-19-related restrictions on the PA of women and established that the PA levels were significantly lower in women during the period targeted by the study [9]. Consistent with our findings, previous study have provided insight into the association between PA, and fatigue [57]. It has been reported that physically active men tend to report little sedentary behavior and the least fatigue, which is parallel to our findings [57]. In addition, the bidirectional contribution of fatigue and PA has been previously reported among healthy adults. Observational studies have demonstrated that fatigue (among other factors) leads to a reduction in PA levels [15]. However, the findings of this study could not demonstrate causation, because PA could influence fatigue and vice versa. Further, gender sensitivity in immunological responses has been reported previously and requires further detection to evaluate its possible role in COVID-induced PA-related fatigue.

The current study has several limitations. The design of this study was cross-sectional, which will not help in understanding the physiological mechanism underlying the cause-and-effect relationship between gender and self-reported health outcomes. Other factors such as psychological and physical fitness may explain the remining variance in fatigue severity and PA. Further experimental research is needed to investigate the physiological mechanism of the differences in fatigue and PA. Additionally, we did not control for women’s menstrual cycles or participants’ potential psychological disorders, which could also affect their fatigue levels [58, 59]. We also did not control for the severity of COVID-19 symptoms, which could impact health status during the recovery period. Furthermore, the follow-up time of the current study was short, and a longer follow-up period might provide healthcare practitioners with insightful information regarding persistent COVID-19 symptoms among survivors. Another limitation was that a subjective measurement was used to assess PA and fatigue. Here, objective measurements for PA, such as actigraphy, could be employed. For fatigue, a list of laboratory tests for the following measures should be considered: blood count, C-reactive proteins, blood glucose, ferritin, B-type natriuretic peptides, and kidney, liver, and thyroid function. This could delineate the specific mechanism underlying the severity of fatigue. Finally, the sample size was small, which may have limited the generalizability of the results. Hence, increasing the sample size is necessary to minimize the risk of type 2 errors.

## Conclusions

The current study illustrated the incidence of lower levels of PA and higher FSS results in women who had recovered from COVID-19. The findings of the present study suggest that fatigue severity has varying degrees of influence on PA, depending on gender sensitivity. Considering the importance of increasing PA levels and decreasing fatigue levels, longitudinal and standardized studies to encourage more PA engagement among female COVID-19 survivors (with treatment interventions to control fatigue) are recommended. Furthermore, PA could play a significant role in modulating fatigue severity. Consequently, interventions aimed at promoting PA in women stand a good chance of addressing disparities in the prevalence of fatigue between men and women.

## Supporting information

S1 Appendix Dietary and vitamin supplement used during COVID-19. (DOCX)

S1 Dataset (DTA)
