# Supplementary material for: A pilot study of the moderating effect of gender on the physical activity and fatigue severity among recovered COVID-19 patients
Source: PLoS One. 2022 Jul 13;17(7):e0269954. doi: 10.1371/journal.pone.0269954 (PMC9278785; doi:10.1371/journal.pone.0269954)
Supplement: S1 Appendix — (DOCX) [file pone.0269954.s001.docx]

Supplementary 1. Dietary and Vitamin supplement used during COVID-19

| Response | **ITEMS** | |
| --- | --- | --- |
| Yes | **Do you use Omega-3 supplements?** | |
| No |  |  |
| 1. I don’t use it 2. Sometime 3. Always | **Vitamin C** | **Did you consume any of the following during the time of COVID infection?** |
| 1. I don’t use it 2. Sometime 3. Always | **Vitamin D** |  |
| 1. I don’t use it 2. Sometime 3. Always | **Vitamin B12 supplements** |  |
| 1. I don’t use it 2. Sometime 3. Always | **Zinc** |  |
